# *De novo* assembly of two chromosome-level rice genomes and bin-based QTL mapping reveal genetic diversity of grain weight trait in rice

**DOI:** 10.3389/fpls.2022.995634

**Published:** 2022-08-22

**Authors:** Weilong Kong, Xiaoxiao Deng, Zhenyang Liao, Yibin Wang, Mingao Zhou, Zhaohai Wang, Yangsheng Li

**Affiliations:** ^1^State Key Laboratory of Hybrid Rice, College of Life Sciences, Wuhan University, Wuhan, China; ^2^Center for Genomics and Biotechnology, Fujian Provincial Key Laboratory of Haixia Applied Plant Systems Biology, Fujian Agriculture and Forestry University, Fuzhou, China; ^3^Key Laboratory of Crop Physiology, Ecology and Genetic Breeding (Jiangxi Agricultural University), Ministry of Education of the People’s Republic of China, Nanchang, China

**Keywords:** rice, genome sequencing, yield improvement, 1,000-grain weight, heterosis loci

## Abstract

Following the “green revolution,” *indica* and *japonica* hybrid breeding has been recognized as a new breakthrough in further improving rice yields. However, heterosis-related grain weight QTLs and the basis of yield advantage among subspecies has not been well elucidated. We herein *de novo* assembled the chromosome level genomes of an *indica/xian* rice (Luohui 9) and a *japonica/geng* rice (RPY geng) and found that gene number differences and structural variations between these two genomes contribute to the differences in agronomic traits and also provide two different favorable allele pools to produce better derived recombinant inbred lines (RILs). In addition, we generated a high-generation (> F_15_) population of 272 RILs from the cross between Luohui 9 and RPY geng and two testcross hybrid populations derived from the crosses of RILs and two cytoplasmic male sterile lines (YTA, *indica* and Z7A, *japonica*). Based on three derived populations, we totally identified eight 1,000-grain weight (KGW) QTLs and eight KGW heterosis loci. Of QTLs, *qKGW-6.1* and *qKGW-8.1* were accepted as novel KGW QTLs that have not been reported previously. Interestingly, allele genotyping results revealed that heading date related gene (*Ghd8*) in *qKGW-8.1* and *qLH-KGW-8.1*, can affect grain weight in RILs and rice core accessions and may also play an important role in grain weight heterosis. Our results provided two high-quality genomes and novel gene editing targets for grain weight for future rice yield improvement project.

## Introduction

Rice is one of the most important food crops in the world, providing food for more than half of the world’s population (Qin et al., 2021). Over the past two decades, multiple high-quality genomes of *indica* and *japonica* subspecies have been assembled, such as Nipponbare (Goff et al., 2002), 9311 (Yu et al., 2002), ZS97 (Zhang et al., 2016a,b), MH63 (Zhang et al., 2016a,b), R498 (Du et al., 2017), IR64 (Tanaka et al., 2020), TN1 (Panibe et al., 2021), Huazhan (Zhang H. et al., 2022), Tianfeng (Zhang H. et al., 2022), etc. Recently, several gap-free reference genomes were completed, namely, ZS97, MH63, PR106, LIMA, LARHAMUGAD, KETANNANGKA, NATELBORO, XL628S, LK638S, J4155S, and HZ (Song et al., 2021; Zhang F. et al., 2022; Zhang Y. et al., 2022). Advances in third-generation sequencing and assembly algorithms have continuously updated the accuracy of the rice pan-genome, revealing some important functionally related structural variations (SVs) and gene copy number variations (gCNVs) (Zhao et al., 2018; Qin et al., 2021; Zhang F. et al., 2022). However, there are dramatically different genetic backgrounds among thousands of rice cultivars, especially between subspecies, including cultivar-specific genes, different alleles of one gene, or gene family expansions (Li et al., 2021). Differences in rice agronomic traits are closely related to these genome variations (Stein et al., 2018; Zhao et al., 2018; Qin et al., 2021). Therefore, the discovery of new genes/alleles related to agronomic traits is inseparable from the comparative analysis of the genomes of elite varieties and the fine mapping in derived populations. For instance, we previously found that the hybrid progeny of the Luohui 9 (*xian*/*indica*) and RPY geng (*geng*/*japonica*) cross had significant heterosis in yield and resistance traits, and multiple recombinant inbred lines (RILs) derived from Luohui 9 X RPY geng aggregated the advantages of both parents (Kong et al., 2022a). Based on the high-density genetic map, we also obtained some QTLs related to plant height, salt stress tolerance, submerged germination, and grain shape (Kong et al., 2021b, 2022a,b; Deng et al., 2022). But the gene number differences and large structural variations between Luohui 9 and RPY geng, and the effects of these variations/differences on traits and heterosis, remain unclear.

Owing to the impacts of human population growth and limited arable land, breeders and scientists faced the challenge of breeding higher yield potential crops. Rice yield is a complex agronomic trait composed of four main factors including effective panicle number, grain number per panicle, seed setting rate and 1,000-grain weight (KGW) (Zuo and Li, 2014). In addition, heterosis refers to the phenomenon that the phenotype of the hybrid progeny surpasses their parents in biomass, yield, growth vigor, resistance, etc., (Birchler et al., 2010). Yield heterosis between *indica* and *japonica* subspecies has been widely used to improve rice yield, causing a worldwide yield revolution (Li et al., 2018). As statistics, hybrid rice shows a 20–30% increase in yield than inbred rice and has effectively solved world food crisis (Xu et al., 2016). Therefore, analyzing the mechanism of rice grain weight (GW) and mining GW-related QTLs and GW-related heterosis loci are important foundations for improving rice yield. Based on different populations and QTL mapping methods, more than 600 QTLs related to grain weight and grain shape have been identified on all 12 chromosomes in rice to date^1^ (Chan et al., 2021), and more than 20 QTLs have been cloned, including *GW2* (Yan S. et al., 2011), *GS3* (Liu et al., 2018), *TGW6* (Ishimaru et al., 2013), *GW6a* (Song et al., 2015), *WTG1* (Huang et al., 2017), *GL7* (Wang et al., 2015), and *gw5* (Wan et al., 2005). In fact, multiple reported QTLs may belong to one QTL, but there are differences in the size of the interval. Meta-analysis of QTLs was used to merge multiple QTLs from different rice genetic populations and to identify consensus and stable QTLs (Arcade et al., 2004; Kong et al., 2020), which narrowed down the confidence intervals of QTLs (Martinez et al., 2016; Zhang et al., 2017). Recently, 339 published GW QTLs merged into 34 Meta-QTLs (MQTLs) in rice (Khahani et al., 2020). The new GW QTLs/genes must be urgently explored in *indica* X *japonica* derived populations to further improve rice yields.

In this study, we performed the chromosome-level *de novo* assembly of the Luohui 9 and RPY geng genomes and characterized their genomic differences in genome-wide scale. Additionally, the KGW traits of derived RIL populations from Luohui 9 X RPY geng in four environments were used for QTL mapping. Two testcross populations derived from the crosses of RILs and Z7A (*japonica*) or YTA (*indica*) were used to explore the heterosis loci of KGW. These results provided a new insight into the diversity mechanism of grain weight in rice.

## Materials and methods

### Materials and sequencing

**FIGURE 1:**
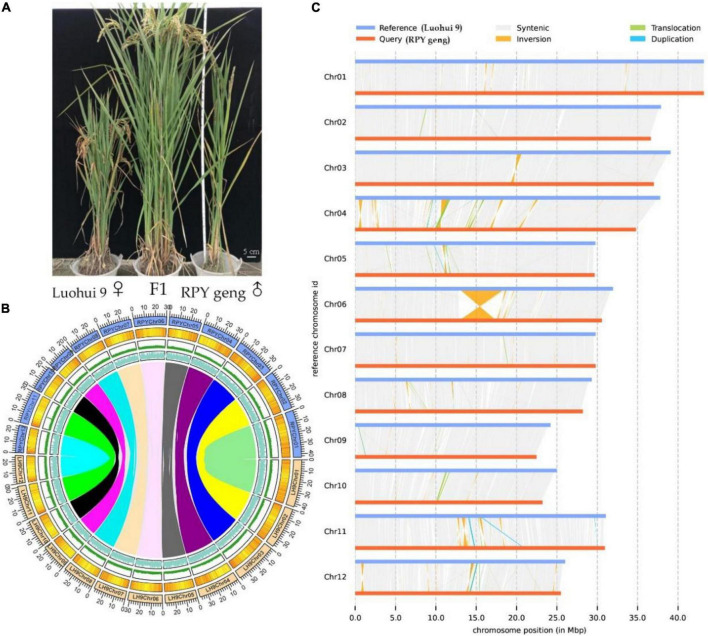
Whole plant phenotype **(A)**, genomic characteristics **(B)**, and large structural variations **(C)** between RPY geng and Luohui 9. The tracks from outside to inside are the chromosome, gene density, repeat sequence density, and GC content and the different color links represent orthologous gene pairs among chromosomes in **(B)**.

The highly homozygous *O. sativa* ssp. *indica*/*xian* (Luohui 9, 2n = 2 × = 24) and *O. sativa* ssp. *japonica*/*geng* (RPY geng, 2n = 2 × = 24) were planted in the field in Wuhan, China, in 2016, These two subspecies have many significant differences in important agronomic traits, including plant height, number of tillers, and heading date, for example, Luohui 9 has excellent agronomic traits, and RPY geng has an ideal plant architecture (Figure 1A).

The genomic DNA of these two subspecies was extracted from young leaves using a modified CTAB method and tested using Qubit Quantitation Starter Kit (Invitrogen, United States) and a 1% agarose gel electrophoresis, respectively. Libraries for Illumina short-read and single-molecule real-time (SMRT) sequencing (Pacific Biosciences, United States) were prepared according to the respective manufacturer’s instructions. The short-read DNA libraries were sequenced by paired-end (2 × 150 bp) method on Illumina HiSeqTM 2,500 (Illumina, United States) and the SMRT sequencing were sequenced on the PacBio RS II platform. In addition, Hi-C reads of DNA of young leaves from F1 progeny of Luohui 9 and RPY geng were sequenced on Illumina HiSeqTM 2,500 paired-end (2 × 150 bp) sequencing according to standard protocol. The total RNA of mixed tissues (root, stem, leaf, and young panicle) was extracted for RNA-seq libraries following the manufacturer’s standard protocol. Then, RNA-seq libraries were sequenced on an Illumina HiSeqTM 2,500 paired-end (2 × 150 bp) and raw reads were filtered using Trimmomatic software as described previously (Kong et al., 2020).

### Genome assembly and annotation

PacBio RSII sub-reads were filtered by the PacBio SMRT-Analysis package including removing the adapters, low quality, and short length reads (parameters: readScore, 0.75; minSubReadLength, 500) and sub-reads after filtering were corrected by Illumina reads using an error correction module embedded in Canu v1.5 (Koren et al., 2017). The high-quality PacBio sub-reads were used for genome contigs assembly by using Canu v1.5 with default parameters. The contig-level genome was polished by Plion with these parameters: –mindepth 10; –changes; –threads 4; –fix bases (Walker et al., 2014). Hi-C data were used to assist in constructing chromosome-level genome assemblies. The Hi-C data were mapped to the contig-level genomes using BWA aligner software (Li and Durbin, 2009). A total of 46.73 Gb clean Hi-C data was mapped to the genome, with a coverage of 116.83 X. These uniquely mapped Hi-C reads were retained for chromosome-level genome assemblies using LACHESIS software with these parameters: CLUSTER MIN RE SITES = 22; CLUSTER MAX LINK DENSITY = 2; CLUSTER NON-INFORMATIVE RATIO = 2; ORDER MIN N RES IN TRUNK = 10; ORDER MIN N RES IN SHREDS = 10 (Burton et al., 2013). Finally, the chromosome-level genome was further improved and corrected by the high-density binmaps genetic map constructed in this study using all maps software according to the method described (Tang H. B. et al., 2015).

Repeat sequence annotation was performed by EDTA with default parameters (Ou et al., 2019). Coding genes were predicted by *de novo*, homolog-based, and transcriptome-based strategies. Augustus v2.4 (Stanke and Waack, 2003), Genscan (Burge and Karlin, 1997), GeneID v1.4 (Alioto et al., 2018), GlimmerHMM v3.0.4 (Majoros et al., 2004), and SNAP (version 2006-07-28) (Korf, 2004) were used for *de novo* prediction. GeMoMa v1.3.1 (Keilwagen et al., 2016) was used for homolog-based prediction. In the transcriptome-based prediction, we used Hisat v2.0.4 and Stringtie v1.2.3 for sequence assembly based on a reference genome (Pertea et al., 2016), and applied TransDecoder v2.0^2^ and GeneMarkS-T v5.1 (Tang S. Y. Y. et al., 2015) for gene prediction; On the other hand, PASA v2.0.2 software (Campbell et al., 2006) was used to perform unigene sequence prediction without reference assembly based on transcriptome sequencing data. Finally, we used the EVM v1.1.1 software (Haas et al., 2008) to integrate all gene prediction results from these three analysis methods. The predicted coding genes were annotated according to alignments against (*E* value 1e–5) databases including GO, KEGG, KOG, TrEMBL, and Nr databases using BLAST v2.2.31 (Altschul, 2012).

### Orthologous clusters analysis and structural variant identification

We extracted the longest-protein sequences from Luohui 9 and RPY geng genomes for orthologous clusters identification using OrthoVenn2, E-value was set 1e–10, and other parameters with default (Xu et al., 2019). Genomic structural variants between Luohui 9 and RPY geng genomes were identified by SyRI (Goel et al., 2019). Luohui 9 genome was used as the reference genome, “nucmer – mum” for sequence alignment, with parameter, -c 100 -l 50 -g 1,000. Then, “delta-filter” was used to filter the comparison results, with parameter, -1 -q -r -i 89 -l 50. Finally, “show-coords,” “syri -c,” and “syri plotsr” steps were done with default parameters.

### QTL mapping of KGW

A 272 RILs and their parents were planted in the Ezhou (30°N, 114°E) Experimental Base of Wuhan University, Wuhan City, Hubei Province in April 2017 (2017EZ) and in April 2018 (2018EZ), the Hybrid Rice Experimental Base of Wuhan University in Lingshui City (18°N, 110°E), Hainan Province in November 2019 (2019LS), and Breeding Experimental Base of Wuhan University Tianyuan Co., Ltd in Hannan District (30°N, 114°E), Wuhan City, Hubei Province in May 2019 (2019HN). Two testcross populations (Z7A-TCF_1_ and YTA-TCF_1_) were developed by crossing RILs (F_14_) with Z7A (*japonica*) and YTA (*indica*) in 2019LS. KGWs of Z7A-TCF_1_, YTA-TCF_1_, and RILs were investigated in 2019HN.

All plants were planted under standard agricultural management practice (Kong et al., 2022a). KGW was surveyed in the above four environments. Each inbred line counted five individual plants, the average KGW value of five individual plants was considered as the KGW value of each inbred line.

The genetic linkage map of 272 RILs including 4,578 bin blocks with the total bin-map distance 2,356.41 cM was previously constructed in our lab (Kong et al., 2022a). The QTL mapping of KGW was analyzed by R/qtl (Arends et al., 2010), the CIM interval mapping method was adopted and the LOD threshold was set by 3.0. The confidence interval was calculated with the function “lodint” (Dupuis and Siegmund, 1999) and the drop value was set to 1.5.

### QTL mapping of KGW heterosis loci

Heterosis related indexes for the KGW trait were calculated by the formulas:


MPH=[F1-(P1+P2)/2]/[(P1+P2)/2]X 100%

BPH=(F⁢1-P⁢1)/P⁢1⁢X⁢ 100%

LPH=(F⁢1-P⁢2)/P⁢2⁢X⁢ 100%

where MPH is middle-parent heterosis, BPH is better-parent heterosis, LPH is lower-parent heterosis, P1 is the high parent, and P2 is the low parent.

The QTL mapping of KGW heterosis related indexes was analyzed by R/qtl (Arends et al., 2010), the CIM interval mapping method was adopted and the LOD threshold was set by 2.5. The confidence interval was calculated with the function “lodint” (Dupuis and Siegmund, 1999) and the drop value was set to 1.5.

## Results and discussion

### *De novo* assembly and annotation of RPY geng and Luohui 9 genomes

A total of 25.1 / 36.5 Gb Illumina short reads with 62 / 91 X coverage of the genome and 15.6 / 19.5 Gb PacBio RSII long reads with 39 / 48 X coverage of the genome of RPY geng / Luohui 9 was obtained (Supplementary Table 1). The long reads were polished by the Illumina paired read and the polished long reads were assembled into contigs by Canu V1.5. After three rounds of contig polish by Pilon v1.22 and Hi-C data correction, we obtained a 383.45 Mb RPY geng genome and a 394.43 Luohui 9 genome, with the contig N50 of 2.81 and 2.84 Mb, respectively (Table 1). Luohui 9 was ∼10.98 Mb larger than the genome of RPY geng. Finally, 96.35 and 96.99 % of contigs were anchored onto 12 pseudo-chromosomes of RPY geng and Luohui 9 based on Hi-C interactions and linkage map from RPY geng x Luohui 9 derived population (Kong et al., 2022a), respectively (Table 1 and Supplementary Figure 1).

**TABLE 1:** Summary of genome assembly and annotation of RPY geng and Luohui 9.

	RPY geng	Luohui 9
**Genome assembly**		
Assembly size (Mb)	383.45	394.43
Number of contigs	684	569
N50 size of contigs (Mb)	2.81	2.84
Anchored contigs (Mb)	369.49	382.54
Anchored contigs (%)	96.35	96.99
Complete assessment of 456 core genes in CEGMA v2.5 (%)	99.56	100
Complete assessment of 238 core genes in CEGMA v2.5 (%)	95.97	97.18
Complete assessment of 1,614 core genes in BUSCO v3.0.2 (%)	98.9	99.1
The long terminal repeat (LTR)-assembly index (LAI)	19.44	19.37
**Genome annotation**		
Percentage of repeat sequences (%)	44.29	46.96
Number of predicted genes	39,255	39,440

The percentages of repeat sequences in the genomes of RPY geng and Luohui 9 were 44.29 and 46.96% based on EDTA with default parameters (Supplementary Table 2). A combination of prediction strategies (*de novo*, homologous based and RNA-seq based) totally identified 39,255 and 39,440 gene models among RPY geng and Luohui 9 genomes, of which 96.81 and 94.75% had at least one annotation result in GO, KEGG, KOG, TrEMBL, or Nr database (Supplementary Tables 3, 4). The results of CEGMA and BUSCO showed that the assembly of RPY geng and Luohui 9 was complete, with more than 95.0% of the core genes. The long terminal repeat (LTR)-assembly index (LAI) of RPY geng and Luohui 9 was 19.44 and 19.37, which is close to the gold genome level (LAI ≥ 20) (Table 1). All the above-mentioned genome indices indicated that the newly assembled genomes of RPY geng and Luohui 9 was of high quality.

### Global genome differences between RPY geng and Luohui 9

RPY geng and Luohui 9 showed obvious differences in yield, grain shape (Deng et al., 2022), plant height (Kong et al., 2022a), and abiotic stress resistances (Kong et al., 2020, 2021a,b,2022b), and the hybrid progeny of RPY geng X Luohui 9 had the excellent heterosis (Figure 1A). These essential agronomic differences are inseparable from the number and structural variation of genes between the two subspecies genomes (Zhao et al., 2018; Qin et al., 2021). Benefiting from the completion of the genomes of RPY geng and Luohui 9, we compared their gene numbers and large structural variations at the genome-wide level and highlighted some genes that have potential impact on agronomic traits. A total of 32,720 orthologous clusters including 32,509 orthologous gene pairs were identified (Supplementary Figure 2 and Figure 1B). Luohui 9 unique orthologous clusters were enriched with multiple essential life GO terms, while RPY geng unique orthologous clusters were enriched with multiple stress-related GO terms (Supplementary Table 5), namely, defense response, cellular response to amino acid stimulus, positive regulation of hydrogen peroxide, as well as response to osmotic stress, suggesting that RPY geng has more tolerance-related genes to abiotic stress than Luohui 9. These results are consistent with our previous findings that RPY geng has stronger resistance to salt stress and cold stress and carries important stress tolerance genes (Kong et al., 2020, 2021a,b,2022b).

We further found 190 inversions, 6,852 translocations, 1,279 (Luohui 9)/1,212 (RPY geng) duplications between RPY geng and Luohui 9 involving 2,234 SV-related genes in Luohui 9 and 1,544 SV-related genes in RPY geng (Supplementary Table 6). Notably, at the position of 12.8–18.6 Mb on Luohui 9 chromosome 6 showed a sequence inversion with a length of about 5.7 Mb compared with the RPY geng genome (Figure 1C) and this inversion has also been reported in previous comparative genomic studies between subspecies (Du et al., 2017; Li et al., 2021; Xie et al., 2021), suggesting that this may be an important structural difference between subspecies. To study the potential roles of these SV-related genes in important agronomic traits, we collected 283 important known genes with functional function verifications (Supplementary Table 7) as query sequences to find homologous genes against SV-related genes by BlastP (value E-10). Totally, 337 SV-related genes were identified as homologous genes of 138 known functional genes belonging to cold tolerance, heat tolerance, salt tolerance, insect resistance, disease resistance, drought tolerance, fertility, grain quality, grain shape, heading date, panicle architecture, nutrient utilization, and panicle architecture (Supplementary Table 8). The above results suggested that the genome-wide number and structural differences play essential roles in the trait differences of RPY geng and Luohui 9, which were consistent with their differential trait characteristics.

### QTLs of KGW

RPY geng and Luohui 9 belonged to *japonica*/*geng* and *indica*/*xian* subspecies, respectively, yield traits of their F1 and many RILs showed obvious over-parent dominance. To resolve yield-related genes, we here conducted trait surveys and linkage analysis of KGW based on the previously constructed high-density genetic map (Kong et al., 2022a).

**FIGURE 2:**
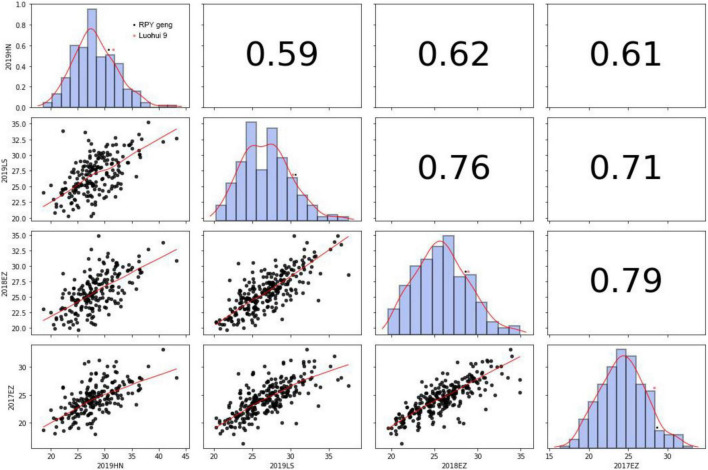
Thousand-grain weight in 2019HN, 2019LS, 2018EZ and 2017EZ.

KGW of RILs were investigated in Lingshui, Hannan, or Ezhou among 2017 – 2019. There was extensive variance of KGW in RIL population while there was a minor difference of KGW between the parents (Figure 2 and Supplementary Table 9). KGW of the RIL population showed a normal distribution with high Pearson coefficients in four different environments and transgressive segregations were observed in the RIL population (Figure 2), which indicated that KGW were controlled by multiple genes and *indica* x *japonica* hybrid breeding strategy can breed high-yielding rice materials.

We totally identified eight KGW QTLs on Chr 2, Chr 3, Chr5, Chr6, Chr8, and Chr10 (Table 2). Of QTLs, *qKGW-8.1* was repeatedly detected in 2017EZ, 2018EZ, and 2019LS. *qKGW-3.1* and *qKGW-3.2* had almost the same interval and *qKGW-5.2* was fully contained by *qKGW-5.1*. These results suggested that these three QTLs had relatively stable effects on KGW in multiple different environments. The remaining QTLs (*qKGW-2.1*, *qKGW-6.1*, and *qKGW-10.1*) were only detected in one specific ecological environment, and were possibly environment-specific KGW QTLs.

**TABLE 2:** Details of thousand-grain weight QTLs.

QTL	Environment	Chr	QTL position	LOD	Size of QTL (Mb)	Phenotypic variation (%)
*qKGW-2.1*	2019LS	Chr 2	22532020–29979073	3.77	7.45	6.18
*qKGW-3.1*	2019HN	Chr 3	8166100–10764126	7.74	2.60	12.28
*qKGW-3.2*	2018EZ; 2019LS	Chr 3	8166100–11054115	6.49; 8.45	2.89	10.41; 13.33
*qKGW-5.1*	2019LS	Chr 5	2493875–5085048	3.1	2.59	5.11
*qKGW-5.2*	2018EZ	Chr 5	3542240–4415859	4.33	0.87	7.07
*qKGW-6.1*	2019_HN	Chr 6	2215678–2577924	4.83	0.36	7.85
*qKGW-8.1*	2017EZ; 2018EZ; 2019LS	Chr 8	4158052–4833970	3.42; 6.2; 4.41	0.68	5.63; 9.96; 6.20
*qKGW-10.1*	2017EZ	Chr 10	13311698–19472799	3.78	6.16	6.2

### Identification and function confirmation of two novel KGW QTLs

**FIGURE 3:**
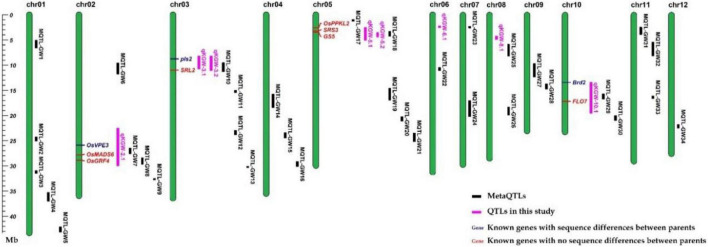
The positions of QTLs, MetaQTLs, and known KGW-related genes.

To distinguish the new TWG QTLs first discovered in this study, 34 Meta-QTLs from 339 original GW QTLs (Supplementary Table 10) and 126 known GW genes (Supplementary Table 11) were collected (Khahani et al., 2020). Except for *qKGW-6.1* and *qKGW-8.1*, the remaining QTLs all had complete or partial overlap with Meta-QTLs, or contained the known KGW genes in these intervals (Figure 3). For example, *qKGW-3.1* and *qKGW-3.2* fully covered *MQTL-GW18* and included *pls2* and *SRL2*. Sequence alignment result showed that *SRL2* had sequence differences in the coding sequence regions (Supplementary Table 12). Similarly, *qKGW-2.1* containing *OsVPE3*, *OsMADS6*, and *OsGRF4*, overlapped *MQTL-GW7* and *MQTL-GW8*; *qKGW-5.1* and *qKGW-5.2* carrying *OsPPKL2*, *SRS3*, and *GS5* overlapped *MQTL-GW18*; *qKGW-10.1* including *FLO7* overlapped *MQTL-GW29*.

**FIGURE 4:**
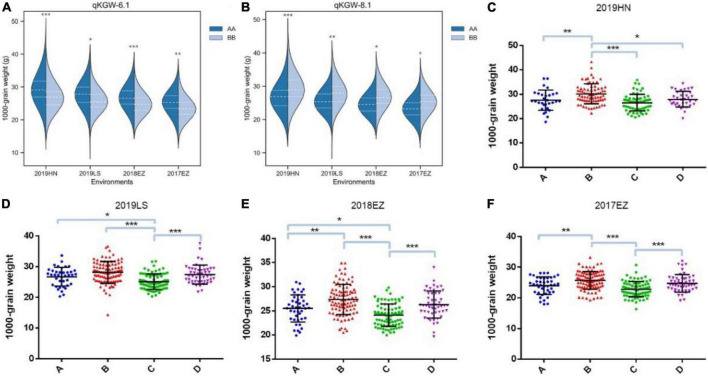
1000-grain weight of different allele combinations of *qKGW-6.1* and *qKGW-8.1.* In **(A)**, **(C–F)**: *qKGW-6.1* AA + *qKGW-8.1* AA, **(B)**: *qKGW-6.1* AA + *qKGW-8.1* BB; **(C)**: *qKGW-6.1* BB + *qKGW-8.1* AA; **(D)**: *qKGW-6.1* BB + *qKGW-8.1* AA.

Therefore, *qKGW-6.1* and *qKGW-8.1* were accepted as novel KGW QTLs. To further confirm the KGW regulation function of *qKGW-6.1* and *qKGW-8.1*, all RILs were divided into different allelic combination based on peak maker genotyping results in genetic map. RPY geng allele (AA) RILs of *qKGW-6.1* showed greater KGW than Luohui 9 (BB) RILs (Figure 4A). Interestingly, *qKGW-8.1* showed the opposite result relative to *qKGW-6.1* (Figure 4B). This suggested that the favorable alleles of these two QTLs are derived from RPY geng and Luohui 9, respectively, and the favorable allele aggregation may enhance KGW of some RILs. As expected, RILs that aggregated favorable alleles (*qKGW-6.1* AA + *qKGW-8.1* BB) had the largest KGW in all tested environments (Figures 4C–F). These results demonstrated that *qKGW-6.1* and *qKGW-8.1* are two new KGW loci that can be used to improve rice yield.

### *Ghd8* has a potential function in regulating rice grain weight

**FIGURE 5:**
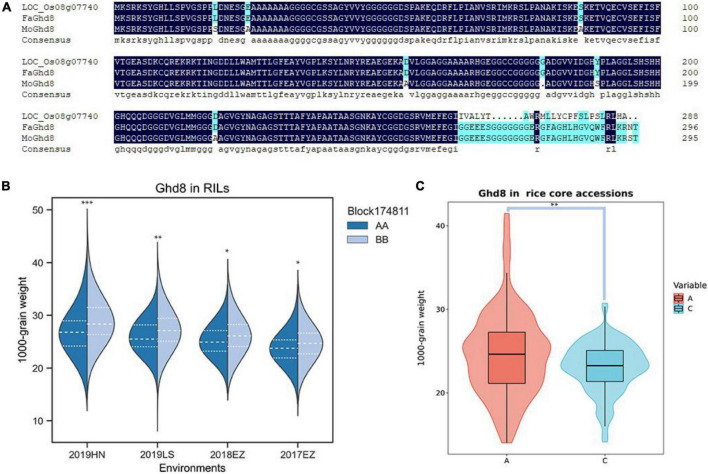
The candidate gene prediction of *qKGW-8.1*. **(A)**. Protein sequence alignment results of Nipponbare, RPYgeng (*FaGhd8*), Luohui 9 (*MoGhd8*). **(B)** The 1,000-grain weight of RPY geng (AA) and Luohui 9 (BB) allele recombinant inbred lines (RILs). **(C)** The 1,000-grain weight of different allele rice core accessions.

*qKGW-8.1* could be detected repeatedly in three environments with phenotypic interpretation rates of 5.11–9.96. However, no known genes directly related to grain weight were found in *qKGW-8.1*. We therefore traversed the 92 gene annotation results in *qKGW-8.1* and tried to correspond them to the phenotypic differences between the parents. We found that *Ghd8* is located within the *qKGW-8.1* interval, a gene reported to be closely associated with heading date and yield (Yan W. H. et al., 2011; Dai et al., 2012), which is consistent with parental heading date differences. Whether in Hainan or Hubei, both parents maintained the heading date difference of more than 10 days. We extracted the Ghd8 protein sequences from our newly assembled genome and sequence alignment revealed multiple sequence variations, including seven amino acid substitutions, one amino acid deletion, and a complex C-terminal amino acid variation (Figure 5A).

To test whether *Ghd8* has a potential effect on grain weight as a pleiotropic gene, we observed grain weight at different alleles in our RILs and in 532 rice core accessions from RiceVarMap v2.0^3^ (Zhao et al., 2021). In RILs, the allele types of *Ghd8* was determined based on a bin maker (Block174811) because *Ghd8* is the only gene within block174811. In 532 rice core accessions, a functional snp (vg0804334484) was found in *Ghd8* gene, containing A, C, and N alleles, and the N allele was eliminated in further phenotypic comparisons due to uncertainty about its base type. We found that different allele RILs or core accessions displayed significantly different KGW, suggesting that *Ghd8* may play a role in KGW regulation (Figures 5B,C).

### KGW heterosis loci

**FIGURE 6:**
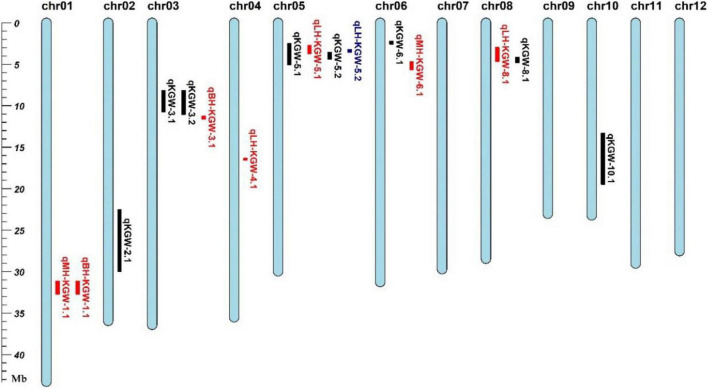
Grain weight heterosis loci in two testcross populations. Black represents QTL loci in recombinant inbred lines (RILs). Red and blue loci represent heterosis-related QTLs of YTA and Z7A testcross population, respectively.

We totally identified two QTLs for KGW BPH, two QTLs for KGW MPH, and four QTLs for KGW LPH (Figure 6 and Supplementary Table 13). Three of the eight heterosis-related QTLs overlapped KGW QTLs: *qLH-KGW-5.1* and *qLH-KGW-5.2* were covered by *qKGW-5.1* and *qLH-KGW-8.1* overlapped with *qKGW-8.1*. Interestingly, *qLH-KGW-8.1* coincided with a reported yield heterosis locus, *RH8* (*rice heterosis 8*) (Li et al., 2016). *Ghd8*, as a major gene in *RH8*, was also located in the *qLH-KGW-8.1* interval. This suggested that *Ghd8* plays an important role in rice yield heterosis. In addition, two GW-related genes were in heterosis-related QTLs, namely, *SRS3* and *GS5* in *qLH-KGW-5.1*, *GS5* in *qLH-KGW-5.2*. Whether these GW-related genes play a role in heterosis remains to be further explored.

## Conclusion

In the present study, we *de novo* assembled genomes of an *indica* rice (Luohui 9) and a *japonica* rice (RPY geng) at the chromosome level and analyzed the KGW trait of their derived RIL populations. We concluded that the substantial genetic diversity of KGW in RILs were closely related to genome variations and allele aggregation difference of KGW QTLs. Importantly, we identified two novel KGW-related QTLs (*qKGW-6.1* and *qKGW-8.1*) and several KGW heterosis loci in three derived population. Based on the genotyping results in RILs and 532 rice core accessions, *Ghd8* in *qKGW-8.1* was presumed to play an important role in GW regulation.

## Data availability statement

The datasets presented in this study can be found in online repositories. The names of the repository/repositories and accession number(s) can be found below: ngdc, PRJCA010706.

## Author contributions

YL and WK conceived and designed the experiments. WK performed genome assembly, analyzed the data, prepared the figures and tables, and wrote the manuscript. XD conducted a field survey of agronomic traits, QTL mapping, and allele genotyping in RILs and rice core accessions. ZW participated in the construction of the recombinant inbred lines and revision of the manuscript. YW provided help in genome annotation. ZL performed repetitive sequence annotation of RPY geng and Luohui 9 genomes. MZ collected Meta-QTLs and known genes performed parts of figures and tables. All authors read and approved the final version of the manuscript.

## Footnotes

1
http://www.gramene.org/

2
http://transdecoder.github.io

3
http://ricevarmap.ncpgr.cn/

## Supplementary material

Supplementary Table 1 The statistic of sequencing data.

Supplementary Table 2 Repeat sequence statistics of Luohui 9 and RPY geng.

Supplementary Table 3 Function annotations of RPY geng genes.

Supplementary Table 4 Function annotations of Luohui 9 genes.

Supplementary Table 5 GO annotation of RPY geng / Luohui 9 unique orthologous clusters.

Supplementary Table 6 SV-related genes in Luohui 9 and RPY geng.

Supplementary Table 7 A 283 important known genes for important agronomic traits.

Supplementary Table 8 Homologous SV-related genes of 138 known functional genes.

Supplementary Table 9 The KGW of the RIL population, RPY geng, and Luohui 9.

Supplementary Table 10 The Meta-QTLs of KGW.

Supplementary Table 11 The known genes of KGW.

Supplementary Table 12 The known genes in KGW QTLs.

Supplementary Table 13 The heterosis-related QTLs of KGW.

## References

[B1] Alioto T. Blanco E. Parra G. Guigó R. (2018). Using geneid to identify genes. *Curr. Protoc. Bioinform.* 64:e56. 10.1002/cpbi.56 30332532

[B2] Altschul S. F. (2012). Basic local alignment search tool (BLAST). *J. Mol. Biol.* 215 403–410. 10.1016/S0022-2836(05)80360-2 2231712

[B3] Arcade A. Labourdette A. Falque M. Mangin B. Chardon F. Charcosset A. (2004). BioMercator: Integrating genetic maps and QTL towards discovery of candidate genes. *Bioinformatics* 20 2324–2326. 10.1093/bioinformatics/bth230 15059820

[B4] Arends D. Prins P. Jansen R. C. Broman K. W. (2010). R/qtl: High-throughput multiple QTL mapping. *Bioinformatics* 26 2990–2992. 20966004 10.1093/bioinformatics/btq565 PMC2982156

[B5] Birchler J. A. Yao H. Chudalayandi S. Vaiman D. Veitia R. A. (2010). Heterosis. *Plant Cell* 22 2105–2112. 20622146 10.1105/tpc.110.076133 PMC2929104

[B6] Burge C. Karlin S. (1997). Prediction of complete gene structures in human genomic DNA. *J. Mol. Biol.* 268 78–94. 9149143 10.1006/jmbi.1997.0951

[B7] Burton J. N. Adey A. Patwardhan R. P. Qiu R. L. Kitzman J. O. Shendure J. (2013). Chromosome-scale scaffolding of de novo genome assemblies based on chromatin interactions. *Nat. Biotechnol.* 31:1119. 10.1038/nbt.2727 PMC4117202 24185095

[B8] Campbell M. A. Haas B. J. Hamilton J. P. Mount S. M. Buell C. R. (2006). Comprehensive analysis of alternative splicing in rice and comparative analyses with *Arabidopsis*. *BMC Genomics* 7:327. 10.1186/1471-2164-7-327 17194304 PMC1769492

[B9] Chan A. N. Wang L. L. Zhu Y. J. Fan Y. Y. Zhuang J. Y. Zhang Z. H. (2021). Identification through fine mapping and verification using CRISPR/Cas9-targeted mutagenesis for a minor QTL controlling grain weight in rice. *Theor. Appl. Genet.* 134 327–337. 10.1007/s00122-020-03699-6 33068118 PMC7813696

[B10] Dai X. Ding Y. Tan L. Fu Y. Liu F. Zhu Z. (2012). LHD1, an allele of DTH8/Ghd8, controls late heading date in common wild rice (*Oryza rufipogon*). *J. Integr. Plant Biol.* 54 790–799. 10.1111/j.1744-7909.2012.01166.x 22963226

[B11] Deng X. Kong W. Sun T. Zhang C. Zhong H. Zhao G. (2022). Bin mapping-based QTL analyses using three genetic populations derived from *indica-japonica* crosses uncover multiple grain shape heterosis-related loci in rice. *Plant Genome* 15:e20171. 10.1002/tpg2.20171 34806841 PMC12806922

[B12] Du H. L. Yu Y. Ma Y. F. Gao Q. Cao Y. H. Chen Z. (2017). Sequencing and de novo assembly of a near complete *Indica* rice genome. *Nat. Commun.* 8:12. 10.1038/ncomms15324 28469237 PMC5418594

[B13] Dupuis J. Siegmund D. (1999). Statistical methods for mapping quantitative trait loci from a dense set of markers. *Genetics* 151 373–386. 9872974 10.1093/genetics/151.1.373 PMC1460471

[B14] Goel M. Sun H. Q. Jiao W. B. Schneeberger K. (2019). SyRI: Finding genomic rearrangements and local sequence differences from whole-genome assemblies. *Genome Biol.* 20:277. 10.1186/s13059-019-1911-0 31842948 PMC6913012

[B15] Goff S. A. Ricke D. Lan T. H. Presting G. Wang R. L. Dunn M. (2002). A draft sequence of the rice genome (*Oryza sativa* L. ssp *japonica*). *Science* 296 92–100. 11935018 10.1126/science.1068275

[B16] Haas B. J. Salzberg S. L. Zhu W. Pertea M. Allen J. E. Orvis J. (2008). Automated eukaryotic gene structure annotation using EVidenceModeler and the program to assemble spliced alignments. *Genome Biol.* 9:22. 10.1186/gb-2008-9-1-r7 18190707 PMC2395244

[B17] Huang K. Wang D. Duan P. Zhang B. Xu R. Li N. (2017). WIDE AND THICK GRAIN 1, which encodes an otubain-like protease with deubiquitination activity, influences grain size and shape in rice. *Plant J.* 5 849–860. 10.1111/tpj.13613 28621888

[B18] Ishimaru K. Hirotsu N. Madoka Y. Murakami N. Hara N. Onodera H. (2013). Loss of function of the IAA-glucose hydrolase gene *TGW6* enhances rice grain weight and increases yield. *Nat. Genet.* 45 707–711. 10.1038/ng.2612 23583977

[B19] Keilwagen J. Wenk M. Erickson J. L. Schattat M. H. Grau J. Hartung F. (2016). Using intron position conservation for homology-based gene prediction. *Nucleic Acids Res.* 44:11. 10.1093/nar/gkw092 PMC4872089 26893356

[B20] Khahani B. Tavakol E. Shariati V. Fornara F. (2020). Genome wide screening and comparative genome analysis for Meta-QTLs, ortho-MQTLs and candidate genes controlling yield and yield-related traits in rice. *BMC Genomics* 21 294–318. 10.1186/s12864-020-6702-1 32272882 PMC7146888

[B21] Kong W. L. Zhang C. H. Qiang Y. L. Zhong H. Zhao G. Q. Li Y. S. (2020). Integrated RNA-seq analysis and Meta-QTLs mapping provide insights into cold stress response in rice seedling roots. *Int. J. Mol. Sci.* 21:14. 10.3390/ijms21134615 32610550 PMC7369714

[B22] Kong W. Deng X. Yang J. Zhang C. Sun T. Ji W. (2022a). High-resolution bin-based linkage mapping uncovers the genetic architecture and heterosis-related loci of plant height in *Indica-japonica* derived populations. *Plant J.* 110 814–827. 10.1111/tpj.15705 35165965

[B23] Kong W. Li S. Zhang C. Qiang Y. Li Y. (2022b). Combination of quantitative trait locus (QTL) mapping and transcriptome analysis reveals submerged germination QTLs and candidate genes controlling coleoptile length in rice. *Food Energy Security* 11:e354.

[B24] Kong W. Sun T. Zhang C. Deng X. Li Y. (2021a). Comparative Transcriptome analysis reveals the mechanisms underlying differences in salt tolerance between *Indica* and japonica rice at seedling stage. *Front. Plant Sci.* 12:725436. 10.3389/fpls.2021.725436 34777413 PMC8578091

[B25] Kong W. Zhang C. Zhang S. Qiang Y. Zhang Y. Zhong H. (2021b). Uncovering the novel qtls and candidate genes of salt tolerance in rice with linkage mapping, RTM-Gwas, and RNA-seq. *Rice* 14:93. 10.1186/s12284-021-00535-3 34778931 PMC8590990

[B26] Koren S. Walenz B. P. Berlin K. Miller J. R. Bergman N. H. Phillippy A. M. (2017). Canu: Scalable and accurate long-read assembly *via* adaptive k-mer weighting and repeat separation. *Genome Res.* 27 722–736. 10.1101/gr.215087.116 28298431 PMC5411767

[B27] Korf I. (2004). Gene finding in novel genomes. *BMC Bioinformatics* 5:9. 10.1186/1471-2105-5-59 15144565 PMC421630

[B28] Li D. Huang Z. Song S. Xin Y. Mao D. Lv Q. (2016). Integrated analysis of phenome, genome, and transcriptome of hybrid rice uncovered multiple heterosis-related loci for yield increase. *Proc. Natl. Acad. Sci. U.S.A.* 113 E6026–E6035. 10.1073/pnas.1610115113 27663737 PMC5068331

[B29] Li F. Gao Y. Wu B. Cai Q. Wang S. (2021). High-quality de novo genome assembly of Huajingxian 74, a receptor parent of single segment substitution lines. *Rice Sci.* 28 109–113.

[B30] Li H. Durbin R. (2009). Fast and accurate short read alignment with Burrows-Wheeler transform. *Bioinformatics* 25 1754–1760. 19451168 10.1093/bioinformatics/btp324 PMC2705234

[B31] Li X. K. Wu L. Wang J. H. Sun J. Xia X. H. Geng X. (2018). Genome sequencing of rice subspecies and genetic analysis of recombinant lines reveals regional yield- and quality-associated loci. *BMC Biol.* 16:12. 10.1186/s12915-018-0572-x 30227868 PMC6145349

[B32] Liu Q. Han R. Wu K. Zhang J. Ye Y. Wang S. (2018). G-protein βγ subunits determine grain size through interaction with MADS-domain transcription factors in rice. *Nat. Commun.* 9:852. 10.1038/s41467-018-03047-9 29487282 PMC5829230

[B33] Majoros W. Pertea M. Salzberg S. (2004). TigrScan and GlimmerHMM: Two open source ab initio eukaryotic gene-finders. *Bioinformatics* 20 2878–2879. 10.1093/bioinformatics/bth315 15145805

[B34] Martinez A. K. Soriano J. M. Tuberosa R. Koumproglou R. Jahrmann T. Salvi S. (2016). Yield QTLome distribution correlates with gene density in maize. *Plant Sci.* 242 300–309. 10.1016/j.plantsci.2015.09.022 26566847

[B35] Ou S. J. Su W. J. Liao Y. Chougule K. Agda J. R. A. Hellinga A. J. (2019). Benchmarking transposable element annotation methods for creation of a streamlined, comprehensive pipeline. *Genome Biol.* 20:275. 10.1186/s13059-019-1905-y PMC6913007 31843001

[B36] Panibe J. P. Wang L. Li J. Li M. Y. Lee Y. C. Wang C. S. (2021). Chromosomal-level genome assembly of the semi-dwarf rice Taichung Native 1, an initiator of Green Revolution. *Genomics* 113 2656–2674. 10.1016/j.ygeno.2021.06.006 34111524

[B37] Pertea M. Kim D. Pertea G. M. Leek J. T. Salzberg S. L. (2016). Transcript-level expression analysis of RNA-seq experiments with HISAT, StringTie and Ballgown. *Nat. Protoc.* 11 1650–1667. 10.1038/nprot.2016.095 27560171 PMC5032908

[B38] Qin P. Lu H. Du H. Wang H. Chen W. Chen Z. (2021). Pan-genome analysis of 33 genetically diverse rice accessions reveals hidden genomic variations. *Cell* 184 3542–3558.e16. 10.1016/j.cell.2021.04.046 34051138

[B39] Song J. M. Xie W. Z. Wang S. Guo Y. X. Koo D. H. Kudrna D. (2021). Two gap-free reference genomes and a global view of the centromere architecture in rice. *Mol. Plant* 14 1757–1767. 10.1016/j.molp.2021.06.018 34171480

[B40] Song X. J. Kuroha T. Ayano M. Furuta T. Nagai K. Komeda N. (2015). Rare allele of a previously unidentified histone H4 acetyltransferase enhances grain weight, yield, and plant biomass in rice. *Proc. Natl. Acad. Sci. U.S.A.* 112 76–81. 10.1073/pnas.1421127112 25535376 PMC4291654

[B41] Stanke M. Waack S. (2003). Gene prediction with a hidden Markov model and a new intron submodel. *Bioinformatics* 19 II215–II225. 10.1093/bioinformatics/btg1080 14534192

[B42] Stein J. C. Yu Y. Copetti D. Zwickl D. J. Zhang L. Zhang C. (2018). Genomes of 13 domesticated and wild rice relatives highlight genetic conservation, turnover and innovation across the genus *Oryza*. *Nat. Genet.* 50 285–296. 10.1038/s41588-018-0040-0 29358651

[B43] Tanaka T. Nishijima R. Teramoto S. Kitomi Y. Hayashi T. Uga Y. (2020). De novo genome assembly of the *Indica* rice variety IR64 using linked-read sequencing and nanopore sequencing. *G3 (Bethesda)* 10 1495–1501. 10.1534/g3.119.400871 32184372 PMC7202035

[B44] Tang H. B. Zhang X. T. Miao C. Y. Zhang J. S. Ming R. Schnable J. C. (2015). ALLMAPS: Robust scaffold ordering based on multiple maps. *Genome Biol.* 16:15. 10.1186/s13059-014-0573-1 25583564 PMC4305236

[B45] Tang S. Y. Y. Lomsadze A. Borodovsky M. (2015). Identification of protein coding regions in RNA transcripts. *Nucleic Acids Res.* 43:10. 10.1093/nar/gkv227 PMC4499116 25870408

[B46] Walker B. J. Abeel T. Shea T. Priest M. Abouelliel A. Sakthikumar S. (2014). Pilon: An integrated tool for comprehensive microbial variant detection and genome assembly improvement. *PLoS One* 9:14. 10.1371/journal.pone.0112963 25409509 PMC4237348

[B47] Wan X. Y. Wan J. M. Weng J. F. Jiang L. Bi J. C. Wang C. M. (2005). Stability of QTLs for rice grain dimension and endosperm chalkiness characteristics across eight environments. *Theor. Appl. Genet.* 110 1334–1346. 10.1007/s00122-005-1976-x 15809851

[B48] Wang Y. Xiong G. Hu J. Jiang L. Yu H. Xu J. (2015). Copy number variation at the GL7 locus contributes to grain size diversity in rice. *Nat. Genet.* 47 944–948. 10.1038/ng.3346 26147619

[B49] Xie X. Du H. Tang H. Tang J. Tan X. Liu W. (2021). A chromosome-level genome assembly of the wild rice *Oryza rufipogon* facilitates tracing the origins of Asian cultivated rice. *Sci. China Life Sci.* 64 282–293. 10.1007/s11427-020-1738-x 32737856

[B50] Xu L. Dong Z. B. Fang L. Luo Y. J. Wei Z. Y. Guo H. L. (2019). OrthoVenn2: A web server for whole-genome comparison and annotation of orthologous clusters across multiple species. *Nucleic Acids Res.* 47 W52–W58. 10.1093/nar/gkz333 31053848 PMC6602458

[B51] Xu S. Z. Xu Y. Gong L. Zhang Q. F. (2016). Metabolomic prediction of yield in hybrid rice. *Plant J.* 88 219–227. 27311694 10.1111/tpj.13242

[B52] Yan S. Zou G. Li S. Wang H. Liu H. Zhai G. (2011). Seed size is determined by the combinations of the genes controlling different seed characteristics in rice. *Theor. Appl. Genet.* 123 1173–1181. 21805338 10.1007/s00122-011-1657-x

[B53] Yan W. H. Wang P. Chen H. X. Zhou H. J. Li Q. P. Wang C. R. (2011). A major QTL, *Ghd8*, plays pleiotropic roles in regulating grain productivity, plant height, and heading date in rice. *Mol. Plant* 4 319–330. 10.1093/mp/ssq070 21148627

[B54] Yu J. Hu S. N. Wang J. Wong G. K. S. Li S. G. Liu B. (2002). A draft sequence of the rice genome (*Oryza sativa* L. ssp *Indica*). *Science* 296 79–92. 11935017 10.1126/science.1068037

[B55] Zhang F. Xue H. Dong X. Li M. Zheng X. Li Z. (2022). Long-read sequencing of 111 rice genomes reveals significantly larger pan-genomes. *Genome Res* 32 853–863. 10.1101/gr.276015.121 35396275 PMC9104699

[B56] Zhang H. Wang Y. Deng C. Zhao S. Zhang P. Feng J. (2022). High-quality genome assembly of Huazhan and Tianfeng, the parents of an elite rice hybrid Tian-you-hua-zhan. *Sci. China Life Sci.* 65 398–411. 10.1007/s11427-020-1940-9 34251582

[B57] Zhang J. W. Chen L. L. Sun S. Kudrna D. Copetti D. Li W. M. (2016a). Building two *Indica* rice reference genomes with PacBio long-read and Illumina paired-end sequencing data. *Sci. Data* 3:160076. 10.1038/sdata.2016.76 27622467 PMC5020871

[B58] Zhang J. W. Chen L. L. Xing F. Kudrna D. A. Yao W. Copetti D. (2016b). Extensive sequence divergence between the reference genomes of two elite *Indica* rice varieties Zhenshan 97 and Minghui 63. *Proc. Natl. Acad. Sci. U.S.A.* 113 E5163–E5171. 10.1073/pnas.1611012113 27535938 PMC5024649

[B59] Zhang X. C. Shabala S. Koutoulis A. Shabala L. Zhou M. X. (2017). Meta-analysis of major QTL for abiotic stress tolerance in barley and implications for barley breeding. *Planta* 245 283–295. 10.1007/s00425-016-2605-4 27730410

[B60] Zhang Y. Fu J. Wang K. Han X. Yan T. Su Y. (2022). The telomere-to-telomere gap-free genome of four rice parents reveals SV and PAV patterns in hybrid rice breeding. *Plant Biotechnol. J.* 10.1111/pbi.13880 35748695 PMC9398309

[B61] Zhao H. Li J. C. Yang L. Qin G. Xia C. J. Xu X. B. (2021). An inferred functional impact map of genetic variants in rice. *Mol. Plant* 14 1584–1599. 34214659 10.1016/j.molp.2021.06.025

[B62] Zhao Q. Feng Q. Lu H. Li Y. Wang A. Tian Q. (2018). Pan-genome analysis highlights the extent of genomic variation in cultivated and wild rice. *Nat. Genet.* 50 278–284. 29335547 10.1038/s41588-018-0041-z

[B63] Zuo J. Li J. (2014). Molecular genetic dissection of quantitative trait loci regulating rice grain size. *Annu. Rev. Genet.* 48 99–118. 25149369 10.1146/annurev-genet-120213-092138

