# Supplementary material for: De novo assembly of two chromosome-level rice genomes and bin-based QTL mapping reveal genetic diversity of grain weight trait in rice
Source: Front Plant Sci. 2022 Aug 22;13:995634. doi: 10.3389/fpls.2022.995634 (PMC9443666; doi:10.3389/fpls.2022.995634)
Supplement: Supplementary file 1 [file Data_Sheet_1.DOCX]

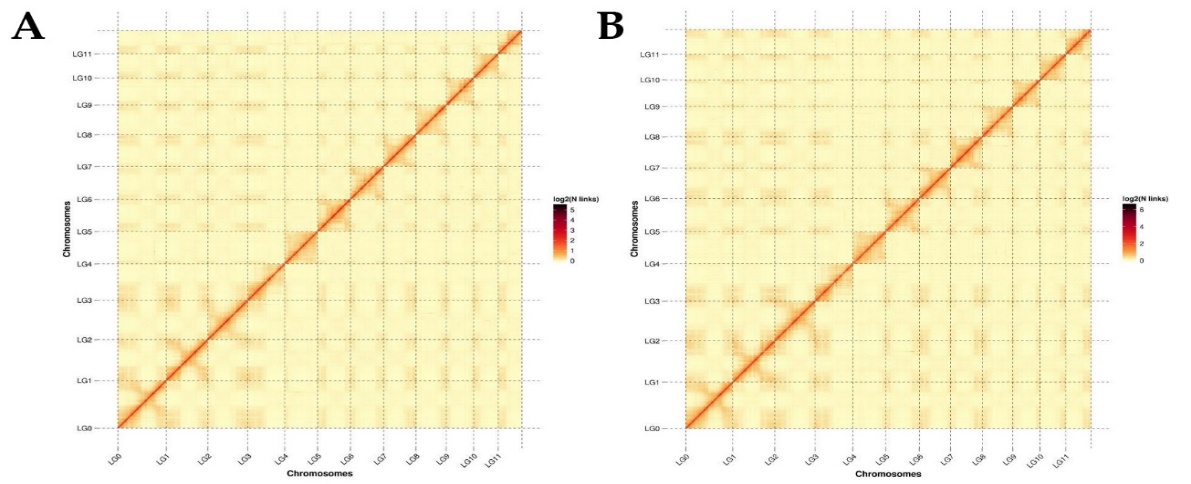
**Figure S1. Bin interactive signal heat map of RPY geng (A) and Luohui 9 (B)**


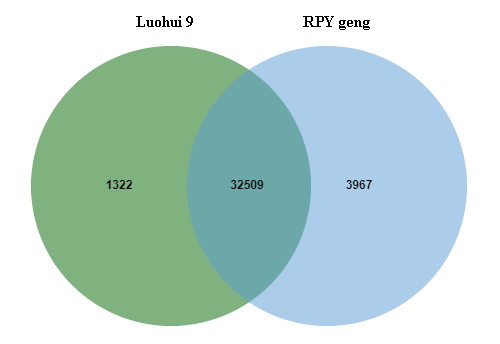


**Figure S2. Veen diagram of RPY geng and Luohui 9 orthologous clusters**
